# A meta-analysis of the effectiveness of gradual versus abrupt smoking cessation

**DOI:** 10.18332/tid/100557

**Published:** 2019-02-13

**Authors:** Jixiang Tan, Lin Zhao, Hong Chen

**Affiliations:** 1 Department of Emergency & Critical Care Medicine, The First Affiliated Hospital of Chongqing Medical University, Chongqing, China; 2 Department of Orthopedics, The First Affiliated Hospital of Chongqing Medical University, Chongqing, China

**Keywords:** smoking cessation, meta-analysis, gradual, abrupt

## Abstract

**INTRODUCTION:**

The aim of this review is to test whether a gradual reduction in smoking results in a superior quit rate compared to abrupt cessation.

**METHODS:**

This review was based on Cochrane methodology for conducting meta-analysis. Only randomized controlled trials were eligible for this review. The participants were adult smokers who were addicted to tobacco, defined as those who smoked at least 15 cigarettes or 12.5 grams of loose-leaf tobacco daily or who had an end-expiratory carbon monoxide concentration of at least 15 ppm. Both groups used an equal amount of nicotine replacement therapy (NRT) before and after quitting smoking. The Review Manager Database (RevMan version 5.3) was used to analyze selected studies.

**RESULTS:**

Three randomized controlled trials involving 1607 patients were included. The prolonged abstinence rate of the gradual cessation group was significantly lower than that of the abrupt group (relative risk, RR=0.77). The result of 7-day smoking cessation rate was also lower in the gradual group (RR=0.76).

**CONCLUSIONS:**

Comparing the combination of NRT and abrupt cessation, the smoking cessation rate of the combination of NRT and gradual cessation is significantly lower. No significant adverse events were found in either group.

## INTRODUCTION

Tobacco smoking is a leading cause of mortality in developed and developing countries^1^. Most smokers, at sometime, have considered quitting. For those smokers interested in quitting, deciding between a gradual decrease or an abrupt quitting method is a common concern^2,3^. Two recommended quit methods in standard cessation programs involve either a gradual reduction of smoking before complete abstinence or abrupt abstinence from cigarettes.

Worldwide guidelines for smoking cessation generally recommend abrupt cessation and do not support a gradual reduction in smoking^4-6^. However, many surveys show that smokers are more likely to choose to stop gradually^2,3,7^. It may be more acceptable to gradually reduce smoking addiction.

Several observational studies found that smoking abstinence rates were higher in smokers who quit abruptly than in those who quit gradually^2,8,9^. However, in these studies, associations may be explained by confounding variables, such as research methods, motivation to quit, self-efficacy, dependence level, or the amount of support received. Those who used the gradual method may have been less likely to receive professional support, since most treatment guidelines do not recommend gradual cessation^10,11^. It is also possible that the lower quit rate in those who quit gradually is explained by adverse self-selection if smokers chose the gradual method only after having failed with the abrupt method. This review aims to test whether an initial gradual reduction in smoking results in a superior quit rate compared with abrupt cessation.

## METHODS

This review was based on Cochrane methodology for conducting meta-analysis^12^.

### Search strategy

The published literature was searched using the electronic databases MEDLINE (1950 to December 2018), AMED (1985 to December 2018), EMBASE (1974 to December 2018), CINHAL (1982 to December 2018), Cochrane Library (2018), CNKI (1994 to December 2018), Scopus and Biomed Central. No language or date restrictions were applied. The Medical Subject Headings (MeSH) and keyword search adopted was ‘smoking cessation’ AND ‘abrupt’ OR ‘gradual’. The unpublished literature was searched using the electronic databases OpenSIGLE (System for Information on Grey Literature in Europe), the WHO International Clinical Trials Registry Platform, Current Controlled Trials, UKCRN Portfolio Database and National Technical Information Service, from their inception to 1 August 2018. Finally, the reference lists of all full-text papers identified as pertinent to the study were reviewed for any unidentified studies.

### Inclusion criteria

Only randomized controlled trials (RCTs) were eligible for this review, with an experimental group that used the gradual method to stop smoking and a control group that received an abrupt method. The participants were adult smokers who were addicted to tobacco, defined as those who smoked at least 15 cigarettes or 12.5 grams of loose-leaf tobacco daily or who had an end-expiratory carbon monoxide (CO) concentration of at least 15 ppm.

Both groups used an equal amount of nicotine replacement therapy (NRT) before and after quitting smoking. In the gradual group, participants aimed to gradually reduce the number of cigarettes smoked within a certain period. The participants of the abrupt group were set a cessation date to quit directly. To ensure the reliability of the results, end-expiratory CO concentrations were only used to check whether the participant quit smoking.

### Study selection

Two authors (TJX, ZL) independently applied the search strategy to selected references from these databases. The titles and abstracts of those articles were reviewed independently. When there was a doubt, the full text was retrieved for further scrutiny. Those two authors independently assessed each full study report to see whether it met the inclusion criteria, and authors were contacted for more information and clarification of data as necessary. Any disagreement was discussed with the senior author (CH), and when consensus could not be reached, that study was excluded. A list of all pertinent papers satisfying these criteria was then constructed by each reviewer, to compile an agreed list of studies.

### Data extraction

A data extraction form was designed and agreed by the authors, and a pilot test of three articles was performed to ensure its consistency. Initially, two authors (TJX, ZL) independently extracted the data, which was later reviewed jointly to produce agreed accurate data. Disagreements were resolved by consensus or consultation with the senior authors. Data extracted included: sample size, study design, subject age, gender, body mass index, highest education, cigarettes per day, minutes to the first cigarette of the day, cigarette type, cigarette dependence scale, age started smoking, prior quit attempts, prior treatments, and confidence in the ability to quit.

### Outcome

The outcome measures were the prolonged and 7-day CO-verified abstinence rates, and other adverse events.

### Quality assessment

To assess the methodological quality of included studies, author (JXT) used the Jadad score^13^, including the proper conduct of randomization, concealment of treatment allocation, the similarity of treatment groups at baseline, clinician blinding, and the description of withdrawals and dropouts. The methodological quality of each trial was scored and ranged from 0 to 5. Any disagreement was resolved by the senior authors.

### Statistical analysis

The Review Manager Database (RevMan version 5.3, Cochrane Collaboration, The Nordic Cochrane Centre, Copenhagen) was used to analyze selected studies. Continuous data for each arm in a particular study were expressed as mean and standard deviation (SD), and the treatment effect as mean differences. Dichotomous data for each arm in a particular study were expressed as proportions or risks, and the treatment effect as relative risk (RR). Missing data were sought from the authors. When this was not possible, or data were missing through loss to follow-up, intention-to-treat principles were used. Statistical heterogeneity was assessed using the value of I^2^ and the result of the chi-squared test. A p-value less than 0.1 and an I^2^ value greater than 50% was considered suggestive of statistical heterogeneity, prompting random effects modelling estimate. Otherwise, a fixed effects approach was used. Conversely, a non-significant chi-squared test result (p≥0.1 and an I^2^ ≤50%) only suggested that there was no evidence of heterogeneity; it did not imply that there was necessarily homogeneity, as there may have been insufficient power to be able to detect heterogeneity. When the data allowed, we performed a subgroup analysis of the trials.

## RESULTS

**Figure 1:**
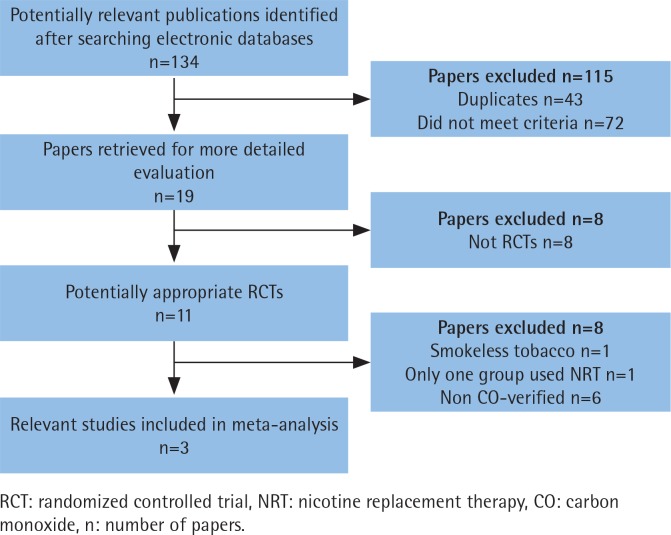
Flowchart of the study selection

**Figure 2:**
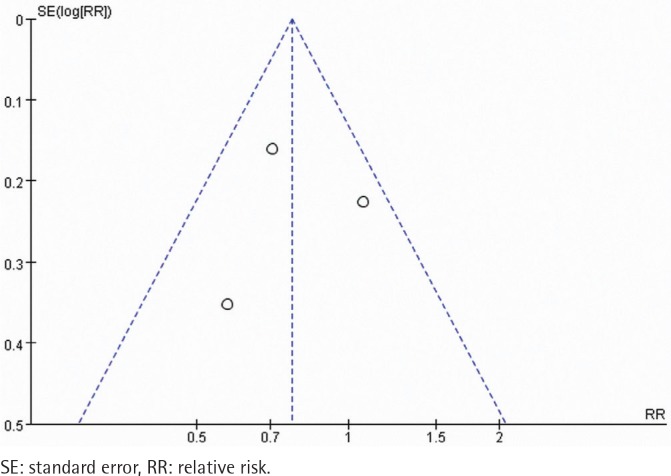
Trials of gradual vs abrupt group: funnel plot of prolonged abstinence rate

A total of 134 abstracts and titles were reviewed. Of these, 3 satisfied the eligibility criteria and were included in the meta-analysis^14-16^. A flowchart is provided in Figure 1. The number of participants included in these RCTs ranged from 314 to 697. A total of 1607 participants were enrolled in the RCTs. The details are shown in Table 1. These studies were relatively well designed with a quality assessment score of 5 (score range 0–5). A funnel plot based on the most frequently cited outcome was broadly symmetrical, indicating minimal publication bias (Figure 2).

**Table 1:** Characteristics of the included studies

*Author*	*Groups*	*Number (n)*	*Age (years)*	*Cigarettes per day (n)*	*Intervention*	*NRT type*	*Prolonged abstinence rate (%)*	*Dropout rate (%)*	*Year*	*Jadad*
Lindson-Hawley^14^	Gradual Abrupt	342 355	49.0 49.0	20.0 20.0	Reduced by 50% in the first week, 75% in the second week, and then to quit. Set a cessation date to quit directly.	Nicotine patches	15.5 22.0	17.3 14.1	2016	5
Hughes^15^	Gradual Abrupt	297 299	48.0±13 48.0±12	23.0±8 23.0±9	Reduced by 25% in the first week, 50% in the second week, 75% in the third week, and then to quit. Set a cessation date to quit directly.	Nicotine lozenges	4.0 7.0	23.6 20.7	2010	5
Etter^16^	Gradual Abrupt	154 160	42.0 44.1	24.0 23.4	Reduced by 50% in four weeks, and then to quit. Set a cessation date to quit directly.	Nicotine gum	20.1 19.4	11.0 12.5	2009	5

NRT: nicotine replacement therapy.

### Prolonged and 7-day CO-verified abstinence rates

**Figure 3:**
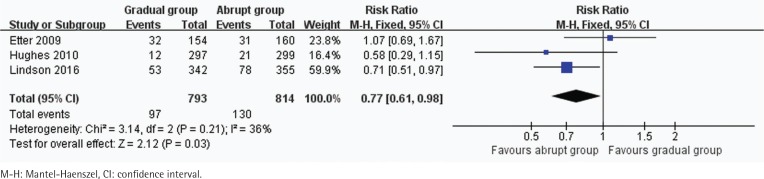
Trials of gradual vs abrupt group: forest plot of prolonged abstinence rate

**Figure 4:**
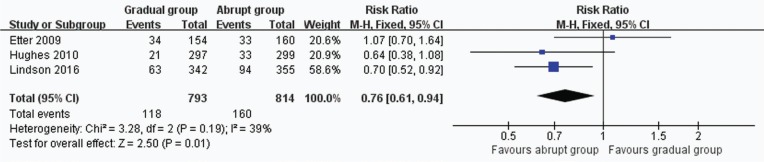
Trials of gradual vs abrupt group: forest plot of 7-day abstinence rate

In all, 3 trials including 1607 participants provided useful data on prolonged and 7-day CO-verified abstinence at the follow-up at 6 or 12 months. The prolonged abstinence in the gradual and abrupt groups were 97 of 793 and 130 of 814 participants,respectively. The prolonged abstinence rate of the gradual group was significantly lower than that of the abrupt group (RR=0.77, 95% CI: 0.61–0.98; p=0.03) (Figure 3). The result of 7-day smoking cessation rate was also lower in the gradual group (RR=0.76, 95% CI: 0.61–0.94; p=0.01) (Figure 4). In addition, the authors of this paper compared all reported adverse events between the groups. However, there were insufficient data to analyze the outcome.

## DISCUSSION

NRT aims to reduce motivation to smoke and the physiological and psychomotor withdrawal symptoms often experienced during an attempt to stop smoking, and thus increase the likelihood of remaining abstinent^17^. Nicotine replacement products are formulated for absorption through the oral or nasal mucosa (chewing-gum, lozenges, sublingual tablets, inhaler/inhalator, spray) or through the skin (transdermal patches). The evidence that NRT helps some people to stop smoking is now well accepted, and many clinical guidelines recommend NRT as a first-line treatment for people seeking pharmacological help to stop smoking^10,11,18-20^. Consequently, NRT plays a very important role in the smoking cessation process.

This meta-analysis showed that the prolonged and 7-day abstinence rates of the combination of NRT and gradual method to quit were significantly lower than the combination of NRT and abrupt method. This is not consistent with results of previous meta-analyses by Lindson-Hawley et al.^21^ who found that the overall rate ratio for abstinence for reduction versus abrupt cessation was 0.94 (95% CI: 0.79–1.13; p=0.51). Although they conducted a subgroup analysis of the data, there was still no significant difference in abstinence rate between the two groups. The meta-analysis included ten trials; it was found that most of these trials were self-reported results. Studies have shown that the self-reported quitting rates are much higher than the smoking cessation rates detected by end-expiratory CO concentration^14-16^. The authenticity of the self-report is relatively low. Other possible reasons for this diversity are inconsistent intervention between trials, and inclusion of non-RCTs and some relatively poor-quality trials.

From the result of this meta-analysis, it can be inferred that smokers who choose the abrupt method are more likely to quit smoking. It is consistent with the population data surveys of Cheong et al.^2^, and West and Brown^22^. Some experts have suggested this is because smokers who chose to delay lose motivation to quit^23-25^. Another explanation could be that the motivation to quit predicts the means by which persons quit and those who are less motivated select gradual cessation^9,26^. It is important to maintain continuous and sufficient motivation to quit smoking. Future studies could examine this possibility.

In this meta-analysis only RCTs were eligible, and these studies were relatively well designed. More than 300 participants were included in each study (ranging from 314 to 697). To ensure the reliability of the results, end-expiratory CO concentrations were only used to check whether the participant had quit smoking, and self-reported studies were excluded. No significant heterogeneity among the different studies existed when prolonged and 7-day abstinence rates were evaluated. To the best knowledge of the authors, the present meta-analysis is the first comparison of nicotine replacement therapy combined with two different smoking cessation methods.

### Limitations

Limitations of this meta-analysis are the low number of articles included while the follow-up time of each article is relatively short. Also, insufficient data are available to enable us to analyze adverse events and perform subgroup analysis of smoking cessation results.

## CONCLUSIONS

Compared with the combination of NRT and abrupt method to quit, the smoking cessation rate of the combination of NRT and gradual method is significantly lower. No significant adverse events were found in either group.

## CONFLICTS OF INTEREST

Authors have completed and submitted the ICMJE Form for Disclosure of Potential Conflicts of Interest and none was reported.
